# Knowledge, Attitudes, and Practices Regarding Dog Bite and Its Management: A Cross-Sectional Study From the Rural Field Practice Area of Indira Gandhi Institute of Medical Sciences (IGIMS), Patna

**DOI:** 10.7759/cureus.74133

**Published:** 2024-11-21

**Authors:** Kumar Amit, Shivani Sinha, Sanjay Kumar Choudhary, Sanjay Kumar, Muhsin Moidu

**Affiliations:** 1 Community Medicine, Indira Gandhi Institute of Medical Sciences, Patna, IND

**Keywords:** animal bite, dog bite, human rabies, rabies, rabies vaccine, who

## Abstract

Introduction: Rabies continues to be a significant concern in India, with lots of deaths reported annually. Approximately one million people undergo post-exposure prophylaxis treatment annually, despite nearly two million dog bites occurring across the country. Notably, dogs are responsible for more than 99% of these bites. Public understanding of rabies treatment remains limited, with many incorrectly believing that it involves 14 injections following a dog bite. However, there is limited awareness about the potential severity of the disease if dog bites are not properly managed. Rabies, an ancient ailment, remains the most dreaded among all contagious diseases.

Aim: This study aimed to assess the knowledge, attitudes, and practices (KAP) of the people residing in rural Patna and also to identify the sociodemographic factors that might influence KAP regarding dog bites and their management.

Methodology: A field-based cross-sectional study was carried out in the rural field practice area of Indira Gandhi Institute of Medical Sciences (IGIMS), Patna. The study population comprised the patients attending the outpatient department (OPD) at the Rural Health Training Centre (RHTC), Maner, during the study period. The study duration was about six months, that is, from November 2023 to April 2024.

Result: The majority (56.4%) of the study population were of the age group 18-40 years. The major bulk of the study population was male, and most of them were illiterate, that is, 67.6%; only 5.2% of the study population were graduate and above. A higher percentage (30.9%) of the study population were farmers or laborers by occupation. About 53.9% of the study population belonged to the upper middle class. A significant difference was seen among age groups in knowledge concerning the cause of rabies (p=0.023), the spread of rabies by dogs (p=0.001), symptoms of rabies such as altered personality (p=0.001), the possibility of rabies cure (p=0.001), and the importance of seeking medical help promptly after a dog bite (p=0.004). Generally, younger patients showed higher awareness and knowledge regarding these aspects compared to older age groups.

Conclusion: The present study showed a significant knowledge gap with respect to cases of dog bite management in rural Patna. Even though awareness of rabies is present, there is an alarming lacuna in this awareness being converted to wound-washing practices and timely seeking of medical care. To decrease the risk associated with dog bites and rabies transmission, structured educational campaigns and community-level interventions are important. These measures can significantly improve rabies prevention in general and rural areas in particular.

## Introduction

Rabies is a viral zoonotic neglected tropical disease (NTD) caused by the rabies virus belonging to the *Rhabdoviridae* family. It is a vaccine-preventable disease occurring in more than 150 countries and territories. An ancient ailment, it still remains the most dreaded among all contagious diseases. More than 99% of all rabies transmission to humans is caused by dog bites [1]. Since there is no effective therapy for rabies, it is extremely important to prevent the disease after animal exposure. Out of all the diagnosed cases of rabies, 14 survivors have been documented with five of those being from India [2].

In Africa and Asia, the poor awareness of the necessity to seek healthcare following a dog bite accounts for the loss of more than 55,000 lives per year [1]. Out of the 11 World Health Organization (WHO) South-East Asian Region (SEAR) countries, eight are endemic for rabies, including India; the exceptions are the Democratic People's Republic of Korea, Maldives, and Timor‑Leste. With the exception of the historically rabies-free islands of Andaman and Nicobar and Lakshadweep, rabies is endemic in all states and union territories of India [3]. A major contributor to the global burden of rabies, India accounts for 36% of the world's rabies-related deaths. Even though there is a lack of accurate data on the burden of rabies in India, rough estimates as per available information suggest that it causes 20,000 deaths every year [1]. Around two million dog bites occur annually in India, but approximately 40% of cases do not receive post-exposure prophylaxis [4]. This is further complicated by the rampant misinformation present among the communities such as 14 injections after being bitten by a dog and the application of oil, herbs, and red chili to wounds [5]. Particularly in rural areas, a large portion of the populace has better faith in traditional remedies than evidence-based practices [6]. Additionally, awareness about the potential severity including its almost universal fatality is very limited.

The importance of community knowledge about rabies cannot be overstated in preventing and controlling rabies [7]. In 2021, India implemented its National Action Plan for Eliminating Dog-Mediated Rabies (NAPRE) to support the global goal of eradicating human deaths from dog-induced rabies by 2030 (zero by 30). The government has mandated that all states must report cases of human rabies, marking a significant step in creating efficient reporting and surveillance systems [8]. This study was done with the aim of assessing the knowledge, attitudes, and practices (KAP) of rural Patna residents regarding dog bites and their management. This information can contribute to designing targeted educational campaigns and interventions to improve community awareness about dog bite prevention and proper management.

## Materials and methods

Study design

A field-based cross-sectional study was carried out in the rural field practice area of Indira Gandhi Institute of Medical Sciences (IGIMS), Patna, after obtaining approval from the Institutional Ethics Committee of IGIMS (approval number: 128//IEC/IGIMS/2023). The study population comprised the patients attending the outpatient department (OPD) at the Rural Health Training Centre (RHTC), Maner, during the study period. The duration of the study was about six months, that is, from November 2023 to April 2024. Using the prevalence rate (p=74%) as taking the awareness of rabies among adults in urban slums near Bangalore, Karnataka, as shown in the study done by Herbert et al. [4], the sample size was calculated using the formula $Sample Size (N) =\frac{Z_{(1-\alpha /2)}(p)(1-p)}{d^{2}}$ where $Z_{(1-\alpha /2)}$ is the standard normal variate (at 5% type I error (p<0.05), it is equal to 1.96), p is the prevalence based on previous studies, and d is the allowed margin of error. Therefore, taking 5% as the allowed margin of error, $Sample Size (N) =\frac{Z_{(1-\alpha /2)}(p)(1-p)}{d^{2}}$ = $\frac{1.96 \times 1.96\times 0.74\times 0.26}{0.05\times 0.05}$= 296. Assuming non-respondents as 10% (i.e., 296×0.10=29.6≈30), the final sample size calculated was 326, rounding off to 330.

Inclusion criteria

Patients over 18 years of age who were willing to participate in the study and had a history of dog bites were included in the study.

Exclusion criteria

Patients under 18 years of age or those unwilling to participate as well as individuals with a history of bites caused by animals other than dogs were excluded from the study.

Data collection

The questionnaire was designed after a literature review and discussion with health workers. It was drafted in English and pre-tested by conducting a pilot study. Cronbach's alpha was calculated and used to check for the reliability of the questionnaire, and a value greater than 0.65 was considered acceptable. The convenience sampling technique was used for the recruitment of the study participants. All the patients who presented to the rural OPD with complaints of a dog bite were approached for willingness to participate in the study. Upon obtaining consent from the participants, the questionnaire was filled out by the primary investigator after explaining the questions in the local language, and thereafter, their responses were recorded.

Statistical analysis

Data entry was done using MS Excel 2015 (Microsoft Corporation, Redmond, Washington, United States). Data analysis was done using IBM SPSS Statistics for Windows, Version 20.0 (Released 2011; IBM Corp., Armonk, New York, United States). Descriptive statistics such as frequencies and percentages were used to describe the study variables. The chi-squared test was applied to test the association between categorical variables, and a p-value less than 0.05 was considered significant. In the situations where the expected counts were found to be less than 5 in more than 20% of the cells, Monte Carlo simulations were utilized to calculate the p-values.

## Results

Sociodemographic characteristics of the participants

A total of 330 patients were included in the present study. The majority of the patients belonged to the age group 18-40 years (56.4%) and were predominantly male (76.7%). There was a high rate of illiteracy (67.6%) among the patients who participated in our study. Socioeconomically, most participants belonged to the upper middle class according to the modified BG Prasad scale (53.9%). A large proportion (30.9%) of patients were employed as farmers or laborers indicating an increased exposure to animals due to outdoor activities (Table 1).

**Table 1:** Sociodemographic profile of the study respondents (n=330)

Demographic variables	Frequency
Age (years)	
18-40	186 (56.4%)
41-60	108 (32.7%)
>60	36 (10.9%)
Gender	
Male	253 (76.7%)
Female	77 (23.3%)
Education	
Illiterate	223 (67.6%)
Primary	23 (7%)
Secondary	43 (13%)
Higher secondary	24 (7.3%)
Graduate and above	17 (5.2%)
Occupation	
Unemployed	97 (29.4%)
Farmer/laborer	102 (30.9%)
Private employee	40 (12.1%)
Govt. employee	1 (3%)
Student	52 (15.8%)
Housewife	38 (11.5%)
Socioeconomic status	
Upper	99 (30%)
Upper middle	178 (53.9%)
Middle	4 (1.2%)
Lower middle	21 (6.4%)
Lower	28 (8.5%)

Table 2 displays the distribution of KAP variables related to rabies cause, spread, prevention, and treatment across different age groups. A significant difference was seen among age groups in knowledge concerning the cause of rabies (p=0.023), the spread of rabies by dogs (p=0.001), symptoms of rabies such as altered personality (p=0.001), the possibility of rabies cure (p=0.001), and the importance of seeking medical help promptly after a dog bite (p=0.004). Generally, younger patients showed higher awareness and knowledge regarding these aspects compared to older age groups. Attitudes of the study population towards specific actions to be taken after being bitten by a dog across different age groups were also analyzed. A significant difference was found in attitudes regarding what to do if bitten by a dog (p=0.001) and the importance of taking a vaccine after a dog bite (p=0.015) among different age groups. However, we found that there was no significant difference in attitudes towards reporting a dog bite to local authorities among the different age groups (p=0.20). Generally, younger patients showed a higher inclination towards taking appropriate actions after a dog bite compared to older age groups. Our study investigated various practices related to rabies prevention and management across different age groups. Significant differences were found among age groups in practices such as knowing where to go for rabies vaccination (p=0.001), washing the wound with soap and water after a dog bite (p=0.008), taking preventive measures to avoid dog bites (p=0.001), and the duration of observation of suspected rabid animals (p=0.001). Younger participants demonstrated more proactive behaviors, such as knowing where to get vaccinated, washing the bite promptly, taking preventive measures, and also adhering to the recommended observation periods for suspected rabid animals, compared to older age groups.

**Table 2:** Knowledge, attitudes, and practices across different age groups among the study participants The chi-squared test was used to test statistical significance. p<0.05 was considered statistically significant a: For each variable in the table, the numbers represent the participants who selected this particular statement among other available options for that question b: Denotes significance values computed using Monte Carlo simulations (based on 10,000 sampled tables) due to the presence of small expected cell counts c: Indicates that more than 20% of cells had expected counts less than 5, warranting caution in the chi-square test's interpretation

Variables^a^	Age (in years)			P-value (chi-squared value)
	18-40	41-60	>60	
Knowledge				
Rabies is caused by a microorganism (4/330)	2 (50%)	1 (25%)	1 (25%)	0.023^b^ (15.27)^c^
Rabies is spread by a dog bite (248/330)	145 (58.46%)	79 (31.85%)	24 (9.67%)	0.001 (32.52)
Altered personality is a symptom of rabies (49/330)	34 (69.38%)	15 (30.61%)	0	0.001 (53.48)
Rabies can't be cured (225/330)	82 (36.44%)	13 (5.77%)	10 (4.44%)	0.001 (32.65)
It is important to seek medical help after a dog bite (261/330)	141 (54.02%)	84 (32.18%)	36 (13.79%)	0.004 (10.84)
Attitudes				
After a dog bite, the wound must be cleaned with soap and water (71/330)	54 (76.05%)	8 (11.26%)	9 (12.67%)	0.001 (30.03)
It's essential to report a dog bite to local authorities (258/330)	144 (55.81%)	80 (31%)	34 (13.17%)	0.20 (11.62)
Vaccination is a necessity after a dog bite (261/330)	139 (53.25%)	89 (34.09%)	33 (12.64%)	0.015 (12.31)
Practices				
Knows where to go for rabies vaccination in their area (256/330)	139 (54.29%)	83 (32.42%)	34 (13.28%)	0.001 (22.04)
Washed the wound with soap and water after a dog bite (52/330)	27 (51.92%)	16 (30.76%)	9 (17.30%)	0.008 (23.72)
As a preventive measure, a safe distance is kept from unfamiliar dogs (121/330)	87 (71.90%)	18 (14.87%)	16 (13.22%)	0.001 (37.97)
Suspected rabid animal should be observed for 10 days (105/330)	52 (49.52%)	52 (49.52%)	1 (0.95%)	0.001 (88.74)

Table 3 shows the distribution of variables related to rabies awareness across different socioeconomic groups. A significant difference was found among different socioeconomic groups regarding the knowledge about rabies. Individuals belonging to the upper middle socioeconomic status had the knowledge that the major cause of rabies was caused by a microorganism, followed by the spread of rabies by dogs. They were aware of the symptoms of rabies such as altered personality, the incurability of rabies, and also the importance of seeking medical help immediately after a dog bite compared to individuals from lower socioeconomic status. The differences were statistically significant with a p-value less than 0.001 for most variables. In our study, we also analyzed the attitudes of the residents towards actions to be taken after being bitten by a dog, reporting the incident to local authorities, and the necessity of taking a vaccine after a dog bite, across different socioeconomic statuses. Results revealed a significant difference in attitudes among socioeconomic groups. Individuals from the upper middle socioeconomic status were more likely to know what to do if bitten by a dog and to understand the importance of taking a vaccine after a dog bite compared to those from lower socioeconomic status, with p-values <0.001 and 0.001, respectively. Also seen were statistically significant differences among socioeconomic groups regarding the belief that it's essential to report a dog bite to local authorities (p<0.001). We also assessed practices related to rabies vaccination like the measures taken after a dog bite, the preventive measures to avoid dog bites, and the period of observation of suspected rabid animals across various socioeconomic statuses. The findings revealed significant differences in practices among different socioeconomic groups. Individuals from the upper middle socioeconomic status were more likely to know where to go for rabies vaccination, to wash with soap and water after a dog bite, to take preventive measures against dog bites, and to observe suspected rabid animals for the recommended period compared to those from lower socioeconomic statuses, with p-values less than 0.05 for all variables.

**Table 3:** Knowledge, attitudes, and practices across socioeconomic groups among the study participants The chi-squared test was used to test statistical significance. p<0.05 was considered statistically significant a: For each variable in the table, the numbers represent the participants who selected this particular statement among other available options for that question b: Denotes significance values computed using Monte Carlo simulations (based on 10,000 sampled tables) due to the presence of small expected cell counts c: Indicates that more than 20% of cells had expected counts less than 5, warranting caution in the chi-square test's interpretation

Variables^a^	Socioeconomic status					P-value (chi-squared value)
	Upper	Upper middle	Middle	Lower middle	Lower	
Knowledge						
Rabies is caused by a microorganism (4/330)	0	3 (75%)	1 (25%)	0	0	0.013^b^ (58.33)^c^
Rabies is spread by a dog bite (248/330)	84 (33.87%)	136 (54.83%)	3 (1.2%)	13 (5.24%)	12 (4.83%)	<0.001^b^ (103.04)^c^
Altered personality is a symptom of rabies (49/330)	20 (40.81%)	21 (42.85%)	0	0	8 (16.32%)	<0.001^b^ (246.14)^c^
Rabies can't be cured (225/330)	95 (42.22%)	111 (49.33%)	2 (0.88%)	1 (0.44%)	14 (6.22%)	0.001 (87.11)
It is important to seek medical help after a dog bite (261/330)	96 (36.78%)	116 (35.15%)	3 (0.91%)	21 (6.36%)	25 (7.58%)	<0.001^b^ (47.35)^c^
Attitudes						
After a dog bite, the wound must be cleaned with soap and water (71/330)	9 (12.67%)	38 (53.52%)	0	6 (8.45%)	18 (25.35%)	<0.001^b^ (107.16)^c^
It's essential to report a dog bite to local authorities (258/330)	95 (36.82%)	123 (47.67%)	2 (0.77%)	14 (5.42%)	24 (9.3%)	<0.001^b^ (61.92)^c^
Vaccination is a necessity after a dog bite (261/330)	96 (36.78%)	112 (46.36%)	3 (1.13%)	14 (5.36%)	27 (10.34%)	0.001^b^ (52.82)^c^
Practices						
Knows where to go for rabies vaccination in their area (256/330)	99 (38.67%)	107 (41.79%)	3 (1.17%)	21 (8.2%)	26 (10.15%)	<0.001^b^ (70.45)^c^
Washed the wound with soap and water after a dog bite (52/330)	17 (32.69%)	22 (42.30%)	0	8 (15.38%)	5 (9.61%)	0.013^b^ (74.46)^c^
As a preventive measure, a safe distance is kept from unfamiliar dogs (121/330)	39 (32.23%)	57 (47.1%)	2 (1.65%)	2 (1.65%)	21 (17.35%)	<0.001^b^ (55.86)^c^
Suspected rabid animal should be observed for 10 days (105/330)	47 (44.76%)	49 (46.66%)	2 (1.9%)	1 (0.95%)	6 (5.71%)	<0.001^b^ (76.58)^c^

## Discussion

In the present study, 56.4% of the participants belonged to the age group of 18-40 years. Similar findings were observed in the study done by Sivagurunathan et al. [9] and Dutta et al. [10]. Additionally, the median age of participants in the study by Ramesh Masthi et al. was 40 years [7], and the mean age of participants in the study by Singh et al. was 41.37 years [11]. With respect to gender distribution, 76.7% of participants were males in our study. Such a finding was also seen in the study done by Dutta et al. (68.8%) [10]. This pattern of gender distribution was different from that observed by Ramesh Masthi et al., Sivagurunathan et al., Singh et al., and Purwar et al. wherein the proportion of both genders was similar among the participants [7,9,11,12]. The male predominance in our study may be due to the fact that adult males are more likely to go for outdoor work [13,14].

Comparing the educational profile of participants of our study with other studies, we found that while 67.6% were illiterate in our study, only a small percentage of participants were illiterate in the studies conducted by Ramesh Masthi et al. [7], Dutta et al. [10], Singh et al. [11], and Purwar et al. [12]. Our findings reflect the low literacy rate prevalent in the rural areas of Bihar. Participants employed as farmers and laborers formed a major chunk of the participants in our study. This trend was also seen in the study conducted by Ramesh Masthi et al. [7] and Sing et al. [11]. Almost half of the study participants in the present study belonged to the upper middle class according to the modified BG Prasad scale. Compared to this, in the study by Dutta et al. [10] and Singh et al. [11], the majority of the participants belonged to the middle class according to the BG Prasad scale [11]. Overall, the sociodemographic profile of participants of the different studies is comparable to our study with few differences which could be attributed to regional differences as none of the studies were conducted in this region.

According to our study, about 145 (58.46%) of patients belonging to the age group 18-40 years knew that dog bite is a cause of rabies. Studies done by Dubey et al. [15] (94.3%) and Dutta et al. (100%) showed a higher proportion of participants sharing this finding [10]. The relatively low awareness in the present study compared to other studies may be due to the low literacy rates observed. Roughly, 68% of participants in the present study knew that rabies is almost an incurable disease. Similar observations were also made by Ramesh Masthi et al. [7], Dubey et al. [15], and Sivagurunathan et al. [9]. This overall increase in awareness regarding dog bites as a source of rabies could be due to increasing dissemination and uptake of information among the general population through activities such as observation of World Rabies Day and awareness campaigns.

Approximately 80% of participants in our study found it important to seek medical help after a dog bite, and a similar observation was also made by Dutta et al. in their study [10]. The attitude regarding seeking medical help after a dog bite was highest among those aged 60 years and belonging to the lower middle socioeconomic class. The better attitude of the elderly population relative to the younger generation could be due to them being exposed to similar incidents in their villages during the course of their life. While most of the population was aware of the anti-rabies vaccine (ARV) for rabies, only about 5% of participants in our study had washed their wounds with soap and water. Similar observations were also made by Dutta et al. [10] and Singh et al. [11]. This reveals a significant gap for future interventions as wound washing is a cornerstone event in the management of animal bites and specifically dog bite wounds.

From the findings of our study and other comparable studies, we see that even though there has been an increase in awareness regarding rabies, only very little of it has translated into actual practices. One important step in this direction can be the introduction of animal bite wounds and rabies in the school curriculum at early stages. Sri Lanka, Malawi, Indonesia, and the Philippines are a few countries that have implemented this and yielded good results [11]. Compared to other modes of intervention, it is also one of the most cost-effective measures. In a study in the Sikkim state of India, it was found that the proportion of students who performed post-exposure precautionary steps increased by 87% following a school-based education intervention [16]. Apart from schools, Village Health and Sanitation Days (VHSNDs) are also an ideal occasion for imparting such practices in rural settings.

Limitations

The findings of this study are bound by a few limitations. The estimation of the sample size for the present study was done on the basis of a study conducted in urban slums which may have introduced a bias. The incidence rate may be an underestimate due to recall bias. Other limitations of this study include its cross-sectional design, which restricts the ability to establish causal relationships between KAP. Since my study was of only six-month duration, the reference period of six months may not be enough to establish the seasonal pattern of dog bites. The reliance on self-reported data may introduce response bias, as participants might underreport negative practices or overstate their knowledge. Additionally, the study's focus on a specific rural area may limit the generalizability of the findings to other regions. Lastly, potential factors such as cultural beliefs and local veterinary services were not explored in depth, which could further influence attitudes and practices regarding dog bites.

Recommendation

This research article effectively examines the KAP surrounding dog bites in a rural community. The findings reveal significant gaps in awareness and appropriate management strategies, highlighting the need for targeted educational programs. To enhance community health outcomes, it is recommended that public health initiatives focus on increasing awareness of dog bite prevention and management. Additionally, involving local health workers in outreach efforts could further improve knowledge and practices in the area. Overall, the study underscores the importance of addressing these issues to mitigate the risks associated with dog bites.

## Conclusions

Inadequate knowledge and lack of appropriate practices combined with a high prevalence of dog bite cases remain a concerning issue requiring the immediate attention of policymakers. In our study, the demographic data indicates a young, predominantly male, illiterate population with a large proportion involved in farming or labor. We found that individuals from the upper middle socioeconomic status were better aware, had a positive attitude, and were more likely to perform practices that would result in rabies prevention than those from lower socioeconomic status. Enhancing rabies prevention in communities through Information, Education, and Communication (IEC) activities and targeted awareness campaigns is essential. A significant step towards this would be to include it in the education curriculum. An educated and aware populace concerning dog bite management would help decrease the incidence of this nearly fatal disease.
